# Emerging Antigenic Variants at the Antigenic Site Sb in Pandemic A(H1N1)2009 Influenza Virus in Japan Detected by a Human Monoclonal Antibody

**DOI:** 10.1371/journal.pone.0077892

**Published:** 2013-10-16

**Authors:** Mayo Yasugi, Ritsuko Kubota-Koketsu, Akifumi Yamashita, Norihito Kawashita, Anariwa Du, Ryo Misaki, Motoki Kuhara, Naphatsawan Boonsathorn, Kazuhito Fujiyama, Yoshinobu Okuno, Takaaki Nakaya, Kazuyoshi Ikuta

**Affiliations:** 1 Department of Virology, Research Institute for Microbial Diseases (RIMD), Osaka University, Suita, Osaka, Japan; 2 Graduate School of Life and Environmental Sciences, Osaka Prefecture University, Izumisano, Osaka, Japan; 3 Kanonji Institute, The Research Foundation for Microbial Diseases of Osaka University, Kanonji, Kagawa, Japan; 4 Department of Genome Informatics, RIMD, Osaka University, Suita, Osaka, Japan; 5 Department of Environmental Pharmacometrics, Graduate School of Pharmaceutical Sciences, Osaka University, Suita, Osaka, Japan; 6 Applied Microbiology Laboratory, International Center of Biotechnology, Osaka University, Suita, Osaka, Japan; 7 Ina Laboratory, Medical & Biological Laboratories Corporation, Ltd., Ina, Nagano, Japan; 8 Department of Medical Sciences, Ministry of Public Health, Muang, Nonthaburi, Thailand; 9 International Research Center for Infectious Diseases, RIMD, Osaka University, Suita, Osaka, Japan; 10 The Japan Science and Technology Agency/Japan International Cooperation Agency, Science and Technology Research Partnership for Sustainable Development (JST/JICA, SATREPS), Tokyo, Japan

## Abstract

The swine-origin pandemic A(H1N1)2009 virus, A(H1N1)pdm09, is still circulating in parts of the human population. To monitor variants that may escape from vaccination specificity, antigenic characterization of circulating viruses is important. In this study, a hybridoma clone producing human monoclonal antibody against A(H1N1)pdm09, designated 5E4, was prepared using peripheral lymphocytes from a vaccinated volunteer. The 5E4 showed viral neutralization activity and inhibited hemagglutination. 5E4 escape mutants harbored amino acid substitutions (A189T and D190E) in the hemagglutinin (HA) protein, suggesting that 5E4 recognized the antigenic site Sb in the HA protein. To study the diversity of Sb in A(H1N1)pdm09, 58 viral isolates were obtained during the 2009/10 and 2010/11 winter seasons in Osaka, Japan. Hemagglutination-inhibition titers were significantly reduced against 5E4 in the 2010/11 compared with the 2009/10 samples. Viral neutralizing titers were also significantly decreased in the 2010/11 samples. By contrast, isolated samples reacted well to ferret anti-A(H1N1)pdm09 serum from both seasons. Nonsynonymous substitution rates revealed that the variant Sb and Ca2 sequences were being positively selected between 2009/10 and 2010/11. In 7,415 HA protein sequences derived from GenBank, variants in the antigenic sites Sa and Sb increased significantly worldwide from 2009 to 2013. These results indicate that the antigenic variants in Sb are likely to be in global circulation currently.

## Introduction

In April 2009, the swine-origin pandemic A(H1N1)2009 virus, A(H1N1)pdm09, emerged, originating from the swine H1 virus in North America and the avian-like swine virus in Europe [1,2]. A(H1N1)pdm09 spread rapidly across the world and is still circulating among humans. One of the factors believed to be contributing to its high transmissibility is the lack of pre-existing immunity in large segments of the global human population [3]. Since its emergence, A(H1N1)pdm09 has remained closely related to one of the earliest viruses isolated, A/California/7/2009, with little change in genetic makeup even in the most variable genes, hemagglutinin (HA) and neuraminidase (NA) [4,5]. The lack of significant antigenic change was reflected in the WHO vaccine formulation decision to recommend the use of an A/California/7/2009-like strain for developing northern hemisphere 2013/14 influenza vaccines [6]. However, even minor changes in the HA molecule may affect receptor binding specificity and antigenicity of the virus [7]. Continued surveillance and antigenic characterization of circulating viruses are therefore crucial to the identification of emerging variants that show significant evolution and that may require the selection of alternative viruses for developing a future vaccine. 

The use of monoclonal antibodies (MAbs) is an established laboratory strategy for characterization of virus strains and their antigenicity [8,9]. In addition to the use of classical murine MAbs (MuMAbs), several methods for the preparation of human MAbs (HuMAbs) have been developed. These range from classical hybridoma methods by cell-cell fusion [10] to more recent methods using transgenic mice [11] and yeast or phage display [12,13]. By using MuMAbs, five classical antigenic sites, Sa (residues 124−125 and 153−165), Sb (residues 187−198), Ca1 (residues 166−170, 203−205 and 235−238), Ca2 (residues 136−142 and 221−222) and Cb (residues 70−75), based on H3 numbering [14], have been identified in the globular head of the HA protein in classical seasonal H1N1 viruses [15]. For A(H1N1)pdm09, homology modeling has revealed similar antigenic sites as described above [16]. In fact, several HuMAbs and MuMAbs have been established against the globular head, including Sa, Sb and Ca2 as above [17-19]. Thus, antigenic sites similar to those in classical seasonal H1N1 could be important for host immune response against A(H1N1)pdm09.

A hybridoma method for HuMAbs was developed previously in our laboratory by fusion of the peripheral blood mononuclear cells (PBMCs) of influenza-vaccinated healthy volunteers with the fusion partner cell line, SPYMEG [20]. In the current study, we established a HuMAb, designated 5E4, against the antigenic site Sb of the HA protein in A(H1N1)pdm09. Using this HuMAb, the potential emergence of HA variants of A(H1N1)pdm09 in Japan was investigated genetically and antigenically for 58 clinical isolates taken from Japanese patients infected with A(H1N1)pdm09 between 2009 and 2011. 

## Materials and Methods

### HuMAb preparation

HuMAbs were prepared as described previously [20]. Briefly, 10 ml of blood was drawn from a healthy volunteer vaccinated with split virus vaccine including HA derived from A/California/7/2009 (The Research Foundation for Microbial Diseases of Osaka University, Osaka, Japan), and then the PBMCs were collected by density gradient centrifugation using Ficoll Pack Plus (GE Healthcare, Uppsala, Sweden). The PBMCs were fused with the SPYMEG cells [20,21] using polyethylene glycol #1500 (Roche Diagnostics, Mannheim, Germany). The fused cells were cultured in Dulbecco’s modified Eagle medium (DMEM) (Invitrogen, Carlsbad, CA) supplemented with 15% fetal bovine serum and hypoxanthine-aminopterin-thymidine. The first screening for MAbs specific for A(H1N1)pdm09 was performed on the basis of reaction with A/Suita/1/2009 by immunofluorescence assay (IFA). The cells in the specific MAb-positive wells were cloned by limiting dilution and then, following a second screening by IFA, the hybridoma cells from positive wells were cultured and expanded in Hybridoma-SFM (Invitrogen). The MAbs were purified from 100 ml of hybridoma culture supernatant by affinity chromatography using HiTrap Protein G HP Columns (GE Healthcare) and then dialyzed into phosphate-buffered saline (PBS) using Slide-A-Lyzer® Dialysis Cassettes (Thermo Scientific, Waltham, MA).

### Virus strains

The following virus strains were used: A/New Caledonia/20/1999 (seasonal H1N1), A/Brisbane/59/2007 (seasonal H1N1), A/Hiroshima/52/2005 (H3N2), A/Uruguay/716/2007 (H3N2), B/Florida/4/2006, and B/Malaysia/2506/2004. Viruses were propagated in Mardin-Darby canine kidney (MDCK) cells or in 9-day-old embryonated chicken eggs.

### Clinical virus isolates

A/Osaka/63/2009 was kindly provided by Osaka Prefectural Institute of Public Health, Osaka. The nasal swabs from patients infected with A(H1N1)pdm09 were kindly provided by Baba Pediatric Clinic and Medical Association of Higashinari-ku, Osaka. For viral isolation, MDCK cells were infected using the nasal swabs in a BD Universal Viral Transport (Becton Dickinson, Franklin Lakes, NJ) and cultured in DMEM/F-12+GlutaMAX^TM^-I (Invitrogen) supplemented with 0.4% bovine serum albumin, antibiotics, and 5 µg/ml acetylated trypsin. Amplified viruses were titrated by a focus-formation assay with MDCK cells. The swab samples collected in December 2009 and January 2011 yielded 30 and 28 clinical isolates, respectively (Tables S1 and S2). 

### Immunofluorescence assay (IFA)

IFA was performed as previously described [21]. Briefly, MDCK cells were inoculated with viruses and incubated for 12 hours at 37°C. After fixation with absolute ethanol, the cells were incubated with the hybridoma supernatant, HuMAb 5E4, or anti-nucleoprotein MuMAb (C43) [22] for 30 minutes at 37°C. The cells were then incubated with FITC-conjugated anti-human IgG (1:500; Jackson ImmunoResearch Laboratories, West Grove, PA) or AlexaFluor 488-labeled anti-mouse IgG (1:1,000; Invitrogen) for 45 minutes at 37°C. The cells were then observed under a fluorescence microscope. 

### Focus-formation assay

The focus-formation assay was performed as previously described [23], but with minor modifications. Briefly, MDCK cells in a 96-well plate were adsorbed with serially 10-fold diluted viruses at 37°C for 1 hour. The cells were then washed with PBS and incubated at 37°C for 12 hours. The cells were fixed and subjected to IFA with C43 MuMAb as described in the “IFA” section above. The cells were then counted and the number of focus-forming units per ml was calculated.

### Transfection with the HA gene

As previously described [24,25], the HA gene of A/Suita/01/2009 was amplified by one-step RT-PCR (Superscript III One-Step RT-PCR System with Platinum *Taq* High Fidelity; Invitrogen) using the following HA primer set: forward, 5’-CGACGGAATTCATGAAGGCAATACTAGTAG-3’, and reverse, 5’-CAGCTCTCGAGTTAAATACATATTCTACACTGTAG-3’. The PCR products were purified using the QIAquick Gel Extraction kit (Qiagen, Hilden, Germany) and inserted into the pGEM-T Easy Vector (Promega, Madison, WI). The plasmid was subcloned into the expression vector pCAGGS/MCSII. The expression plasmids were then transfected into human embryonic kidney 293T cells with lipofectamine 2000 (Invitrogen) according to the manufacturer’s instructions. Expression was confirmed by IFA using an anti-HA MuMAb (C179; Takara, Shiga, Japan).

### IgG isotyping

IgG isotyping was carried out as described previously [21]. ELISA microplates (MaxiSorp; Nunc, Penfield, NY) were coated overnight at 4°C with goat anti-human IgG (Jackson ImmunoResearch Laboratories) in 0.05 M sodium bicarbonate buffer (pH 8.6). After washing with PBS including 0.1% Tween-20, the wells were blocked with 0.5% bovine serum albumin in PBS for 1 hour at 37°C. After washing again, the wells were incubated with hybridoma supernatants or control serum for 2 hours at 37°C. Following further washing, the wells were incubated with HRP-conjugated anti-human IgG1, IgG2, IgG3 or IgG4 (SouthernBiotech, Birmingham, AL) for 1 hour at 37°C. The wells were washed five times followed by incubation with tetramethylbenzidine peroxidase substrate (KPL, Gaithersburg, MD) at room temperature in the dark. After 20 minutes, the reaction was stopped with 2N H_2_SO_4_ solution. Color development was read at 450 nm in an ELISA Photometer (Biotek ELISA Reader; Biotek, Winooski, VT). All samples were run in triplicate.

### Hemagglutination-inhibition (HI) assay

First, viral titers were determined by a hemagglutination assay. Briefly, the viruses were serially 2-fold diluted with PBS and the dilutions were mixed with 0.7% (v/v) guinea pig red blood cells (NIPPON BIOTEST LABO, Tokyo, Japan). After incubation at room temperature for 1 hour, hemagglutination units (HAUs) were estimated. Next, HI titration was performed as follows. Purified HuMAb 5E4 at a concentration of 100 µg/ml (reciprocal Ab dilution = 1) were serially 2-fold diluted and mixed with 8 HAU per 50 µl of viral sample. After incubation at 37°C for 1 hour, the mixtures were further incubated with 0.7% (v/v) guinea pig red blood cells for 1 hour at room temperature. The maximal reciprocal MAb dilution that completely inhibited hemagglutination was designated the HI titer. One hundred microliters of ferret anti-A/California/7/2009 serum (kindly provided by Dr. M. Tashiro, National Institute of Infectious Diseases, Japan) was mixed with 300 µl of receptor destroying enzyme (RDE; Denka Seiken, Tokyo, Japan) to eliminate any non-specific inhibitors and incubated at 37°C for 18 hours. This step was followed by heat inactivation at 56°C for 30 minutes, dilution in PBS (final volume = 1 ml), and removal of nonspecific agglutinator by absorbing with test erythrocytes for 1 hour at room temperature [26]. The HI assay was then performed as described above (minimal reciprocal Ab dilution = 10).

### Viral neutralization (VN) assay

The VN test was carried out as described previously [21] but with minor modifications. MAbs at a concentration of 100 µg/ml were serially 4-fold diluted with Minimum Essential Medium (Invitrogen) and incubated with 200 focus-forming units of virus isolates at 37°C for 1 hour. Then, MDCK cells were adsorbed to the mixtures at 37°C for 1 hour. After incubation for 12 hours, the cells were fixed and subjected to IFA. The maximal reciprocal MAb dilutions that inhibited 50% of viral growth were designated the VN_50_ titer. Ferret anti-A/California/7/2009 serum was prepared as described in “HI assay”.

### Selection of escape mutants

Escape mutants were selected by culturing A/Suita/1/2009 or A/Osaka/63/2009 in the presence of MAb as described previously [27], with minor modification. Viruses were incubated with serially 10-fold diluted MAb (to give final concentrations of 0.0025 to 2.5 µg/ml) at 37°C for 1 hour. MDCK cells in a 24-well plate were then inoculated with the mixtures. After culturing for 72 hours in DMEM/F-12+GlutaMAX^TM^-I supplemented with 0.4% bovine serum albumin, antibiotics, and 2 µg/ml acetylated trypsin, the supernatants were collected and subjected to VN and HI assays. Those viral samples that showed more than 4-fold reduction of VN_50_ and HI titer were subjected to direct sequencing analysis of their entire HA gene.

### Sequencing of HuMAb variable regions

Total RNA, extracted from the hybridoma using an RNeasy Mini kit (Qiagen), was subjected to reverse transcription-polymerase chain reaction (RT-PCR) using a PrimeScript RT reagent kit (Takara) with an oligo (dT) primer. The coding regions of the H- and L-chains of HuMAb were amplified by PCR with the following primers: 5’-ATGGAGTTTGGGCTGAGCTGGGTT-3’ (H-chain-forward)/5’-CTCCCGCGGCTTTGTCTTGGCATTA-3’ (H-chain-reverse) and 5’-ATGGCCTGGRYCYCMYTCYWCCTM-3’ (L-chain-forward)/ 5’-TGGCAGCTGTAGCTTCTGTGGGACT-3’ (L-chain-reverse), respectively. PCR products were ligated into pGEM-T Easy Vector (Promega) and their sequences were determined and analyzed using a BigDye Terminator v3.1 Cycle Sequencing kit and an ABI Prism 3100 Genetic Analyzer (Applied Biosystems, Carlsbad, California). The sequences were then analyzed and used to search the NCBI database using IgBLAST software (http://www.ncbi.nlm.nih.gov/igblast/).

### Molecular modeling

The HA structure was constructed using Molecular Operating Environment software (Chemical Computing Group, Motreal, Canada) based on the crystal structure of A/California/4/2009 (PDB ID: 3LZG) [28].

### Antigen-capture ELISA (AC-ELISA)

First, the amount of viral antigen (wild-type viruses and escape mutants of A/Suita/1/2009 and A/Osaka/63/2009) was confirmed. ELISA microplates (MaxiSorp; Nunc) were coated with a mouse anti-HA antibody (C179; 200 µg/ml in PBS) overnight at 4°C. The wells were then blocked with skim milk (5% in PBS) for 2 hours at 37°C. After washing with PBS, the wells were incubated with viruses (2-fold serial dilutions) for 1 hour at 37°C. They were then washed five times in PBS and incubated with A(H1N1)pdm09-vaccinated human serum (1:100) for 1 hour at 37°C. After washing further five times in PBS, the wells were incubated with HRP-conjugated goat anti-human IgG (1:2000; Jackson ImmunoResearch Laboratories) for 1 hour at 37°C, washed five times in PBS, and then incubated with tetramethylbenzidine peroxidase substrate (Sigma, St. Louis, MO) at room temperature in the dark. After 30 minutes, the reaction was stopped by adding 2N H_2_SO_4_ solution. Color development was measured at 450 nm in an ELISA Photometer (Biotek ELISA Reader; Biotek). Next, an AC-ELISA was performed using 5E4. Coating, blocking, incubation with the HRP-conjugated Ab, and color development were performed/measured as described above. Each of viruses was diluted with PBS to apply the same amount of viral antigen. Antigen was detected by HuMAb 5E4, which was added to the wells at concentrations of 10 or 100 µg/ml for 1 hour at 37°C. HuMAb D23-1G7C2 against dengue virus [29] was used as a control.

### RDE avidity assay

A 10% solution (v/v in PBS) of guinea pig erythrocytes was treated with various concentrations (0.5 to 32 µg/ml) of *Vibrio cholerae* RDE (Sigma) for 1 hour at 37°C as described by Hensley et al. [30]. The cells were then washed and diluted again in PBS to yield a 2% (v/v) solution. An aliquot of each 2% erythrocyte solution (25 µl) was added to an aliquot of influenza virus (4 HAU/100 µl) to yield a final volume of 125 µl. The mixture was then incubated for 1 hour at room temperature before the degree of agglutination was measured. Data were expressed as the maximal concentration of RDE that still allowed full agglutination.

### Treatment with α2,3-sialidase

Treatment with α2,3-sialidase was performed as previously described [25]. Briefly, 100 µl of a 10% (v/v) solution of guinea pig red blood cells were treated with 10 U of α2,3-sialidase (New England Biolabs, Ipswich, MA) at 37°C for 18 hours and then washed with PBS. The erythrocyte suspension was then diluted to 0.7% (v/v) and hemagglutination titers were measured as described above.

### Direct sequencing analysis

Viral RNA was extracted with the QIAamp Viral RNA Mini Kit (Qiagen) and subjected to one-step RT-PCR (SuperScript III with platinum Taq HiFi, Invitrogen) with the HA forward primer 5’-CGACGATGAAGGCAATACTAGTAG-3’ and reverse primer 5’-CAGCTTTAAATACATATTCTACACTGTAG-3’ [25]. The PCR products were purified with the QIAquick PCR Purification kit (Qiagen). After electrophoresis, the discrete band was extracted using the QIAquick Gel Extraction kit (Qiagen) and sequenced. 

### Measurement of selection pressure

Rates of nonsynonymous and synonymous substitutions per site (*d*_N_ and *d*_S_, respectively) were estimated for each 51 bp window (sliding in 3 bp increments) and for each epitope region by the Miyata-Yasunaga method [31] using Sqdif Plot [32]. *d*_N_>*d*_S_ and *d*_N_<*d*_S_ indicate positive and negative selection, respectively [33,34]. 

### Sequencing alignment

The influenza amino acid sequences and annotations (influenza.faa and influenza_aa.dat, respectively) were downloaded from the FTP site of Influenza Virus Resource [35] on April 11, 2013. A(H1N1)pdm09 HA sequences were collected by referring to the annotation. The HA sequences were aligned using the MAFFT program [36], after removing truncated sequences more than 30 amino acids long, according to the BLASTP search [37] results against the HA sequence of A/Wakayama/57/2009. A neighbor-joining phylogenetic tree was constructed using the MEGA5 program [38,39] and used to identify a cluster of A(H1N1)pdm09 sequences. For the following analysis, we used the sequences that appeared in this A(H1N1)pdm09 cluster, after aligning them using the MAFFT program.

### Shannon index calculation

The amino acid diversity at each epitope was estimated according to the Shannon index [40] using the following formula:


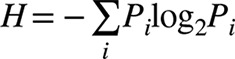


in which H is the Shannon index and P_i_ is the proportion of the *i*-th variant of the epitope.

### Statistical analyses

The Mann-Whitney U test was used to analyze the HI and VN_50_ titers. The Steel-Dwass method was used to analyze the proportion of mutant sequences. A *P*-value of <0.05 was considered significant.

### Accession numbers

The GenBank (http://www.ncbi.nlm.nih.gov/GenBank/) accession numbers for the immunoglobulin genes discussed in this paper are as follows: IgG-V_H_ in clone 5E4 (AB777475) and IgG-V_L_ in clone 5E4 (AB777476). The accession numbers analyzed for viruses in this study are as follows: AB675046 to AB675075 and AB777447 to AB777474.

### Ethics statement

Human materials were collected using protocols approved by the Institutional Review Boards of the Research Institute for Microbial Diseases, Osaka University, Japan (#19-8-6 and #21-5-4). Written informed consent was obtained from the participants.

## Results

### Establishment of HuMAbs against A(H1N1)pdm09

**Figure 1:**
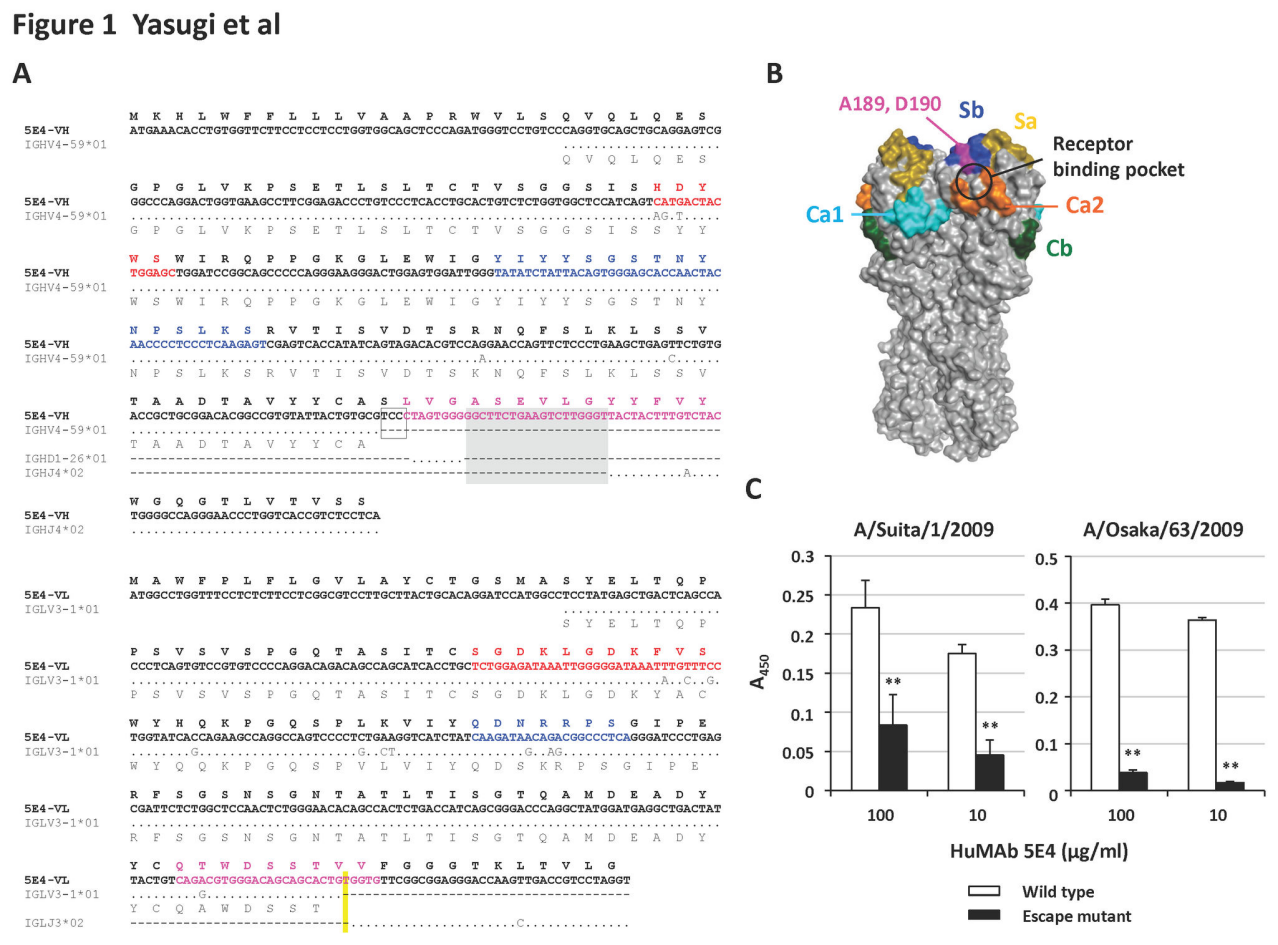
Characterization of HuMAb 5E4. (A) Nucleotide and amino acid sequences of the V_H_ and V_L_ of HuMAb 5E4. The closest germline sequences to the V_H_ and V_L_ of HuMAb 5E4 in the NCBI database, found by IgBLAST software, were aligned. Complementarity-determining regions (CDRs) are indicated in red, blue and pink (CDRs 1, 2 and 3, respectively). V-D and D-J junctions in VH and V-J junctions in VL are highlighted in white, gray and yellow, respectively. (B) Epitope region of HuMAb 5E4 in structural model of HA in A(H1N1)pdm09. A trimer complex is shown in surface representation with the antigenic sites highlighted: Sa in yellow, Sb in blue, Ca1 in cyan, Ca2 in orange and Cb in green. The epitope regions, A189 and D190, are shown in pink. The receptor binding pocket is circled. (C) AC-ELISA using wild-type viruses (open bars) and escape mutants (solid bars) of A/Suita/1/2009 (left panel) and A/Osaka/63/2009 (right panel). MuMAb C179 and HuMAb 5E4 were used as the coating and detecting antibodies, respectively. All data are represented as the means ± s.d. of three independent experiments. Asterisks denote *P*<0.01.

PBMCs were prepared from a healthy volunteer 2 weeks after vaccination with the HA split vaccine, which included the A/California/7/2009 strain, and were used for the fusion with SPYMEG cells. After screening for HuMAb specificity to influenza viruses, the cells in the specifically MAb-positive wells were cloned by limiting dilution. Ultimately, a hybridoma clone was established that produced a HuMAb, designated 5E4, against A(H1N1)pdm09, which did not react with seasonal influenza A (A/New Caledonia/20/1999 and A/Brisbane/59/2007 for H1N1 and A/Hiroshima/52/2005 and A/Uruguay/716/2007 for H3N2) or influenza B viruses (B/Florida/4/2006 and B/Malaysia/2506/2004), according to IFA (data not shown). This HuMAb was shown to recognize the HA protein expressed in transfected 293T cells (data not shown). IgG isotyping revealed that HuMAb 5E4 was IgG1. The V_H_ and V_L_ regions of the HuMAb were sequenced and analyzed with respect to the closest germline sequences using IgBLAST software in the NCBI database (Figure 1A). In addition, 5E4 showed strong activity in the VN and HI assays (Table 1). 

**Table 1:** Characterization of escape mutants of 5E4.

Virus	HI assay^*^	VN assay^†^	Amino acid change	RDE avidity assay (µg/ml)^£^
A/Suita/1/2009	16	64		4
A/Suita/1/2009-escape	1>	16	A189T^‡^	2
A/Osaka/63/2009	256	1024		2
A/Osaka/63/2009-escape	1>	1	D190E^‡^	4

^*^ The results are expressed as the highest dilution of purified HuMAb that completely inhibited hemagglutination.

^†^ The results are expressed as the highest dilution of purified HuMAb that inhibited 50% of viral growth *in vitro*.

^‡^ Based on H3 numbering.

^£^ The results are expressed as the highest concentration of RDE that showed hemagglutination.

To define the 5E4 epitope more precisely, escape mutants were obtained from A/Suita/1/2009 and A/Osaka/63/2009 by co-culturing viruses with 5E4 (Table 1). Six out of twenty-four samples in the first trial with A/Suita/1/2009 showed viral amplification. However, they were neutralized by 5E4 and the HA gene was not mutated. The amplified six samples were then incubated with 5E4 in a second trial. Of 144 wells (24 wells per sample), 48 wells showed viral amplification. However, they were neutralized by 5E4 and the HA gene was not mutated again. Then, we randomly selected six specimens from the second trial and used them in a third trial. Sixty out of one hundred and forty-four samples showed viral amplification. Of these, three showed reduced HI and VN activity in the presence of 5E4 (an 8–16-fold reduction in HI activity and a 4–16-fold reduction in the VN_50_ titer compared with the wild-type virus). When we sequenced the entire HA gene from each of these samples, we found that all harbored an A189T substitution (based on H3 numbering) [14]. Fifteen out of twenty-four A/Osaka/63/2009 samples showed reduced HI and VN activity in the presence of 5E4 (a 32–256-fold reduction in HI activity and a 256–1,024-fold reduction of VN_50_ titer compared with the wild-type virus). The entire HA gene was sequenced in 9/15 samples, and all harbored a D190E substitution. Viruses incubated in the absence of 5E4 showed no amino acid substitutions. These substitutions were positioned within the antigenic site Sb (amino acid position 187 to 198) and in close proximity to the receptor binding domain (Figure 1B) [28,41,42]. 

To classify these mutations as either antigenic or adsorptive, we performed AC-ELISA using one escape mutant from each of the viruses shown in Table 1. Antigenic mutations significantly reduce antibody binding to the target epitope, whereas adsorptive mutations increase the affinity of the virus for the host cell [30]. Compared with the wild-type virus, both of A/Suita/1/2009 and A/Osaka/63/2009 escape mutants showed significantly reduced binding affinity in the AC-ELISA (Figure 1C). Each of viruses was not captured by control HuMAb D23-1G7C2 against dengue virus [29] (Figure S1). Thus, these escape mutants result from antigenic mutations. This was confirmed by treating erythrocytes with RDE, which removes terminal sialic acid residues (the ligand for HA). Compared with the parental virus, adsorptive mutants increase the agglutination of RDE-treated erythrocytes [17]. We found that the escape mutants and the wild-type viruses agglutinated RDE-treated erythrocytes to a similar level (Table 1). These results indicate that the mutants from A/Suita/1/2009 and A/Osaka/63/2009 are antigenic mutants, and that HuMAb 5E4 recognizes amino acids within the antigenic site Sb. Changes in the HA amino acid sequence may affect the receptor specificity of influenza viruses [7]. Thus, we examined the receptor specificity of the mutant viruses. HA titers were measured using guinea pig erythrocytes treated with α2,3-sialidase. As shown in Table 2, α2,3-sialidase treatment did not alter the HA titers. By contrast, α2,3-tropic egg-adapted A/Osaka/63/2009 did not agglutinate erythrocytes after treatment with α2,3-sialidase. These results suggest that, like the wild-type, the two escape mutants are α2,6-tropic.

**Table 2:** Hemagglutination titers using guinea pig erythrocytes treated with or without α2,3-sialidase.

α2,3-sialidase	–	+
A/Suita/1/2009	8	8
A/Suita/1/2009-escape	4	4
A/Osaka/63/2009	4	4
A/Osaka/63/2009-escape	2	2
Egg-passaged A/Osaka/63/2009	64	2>

### Antigenic variants of Sb in Japan

**Figure 2:**
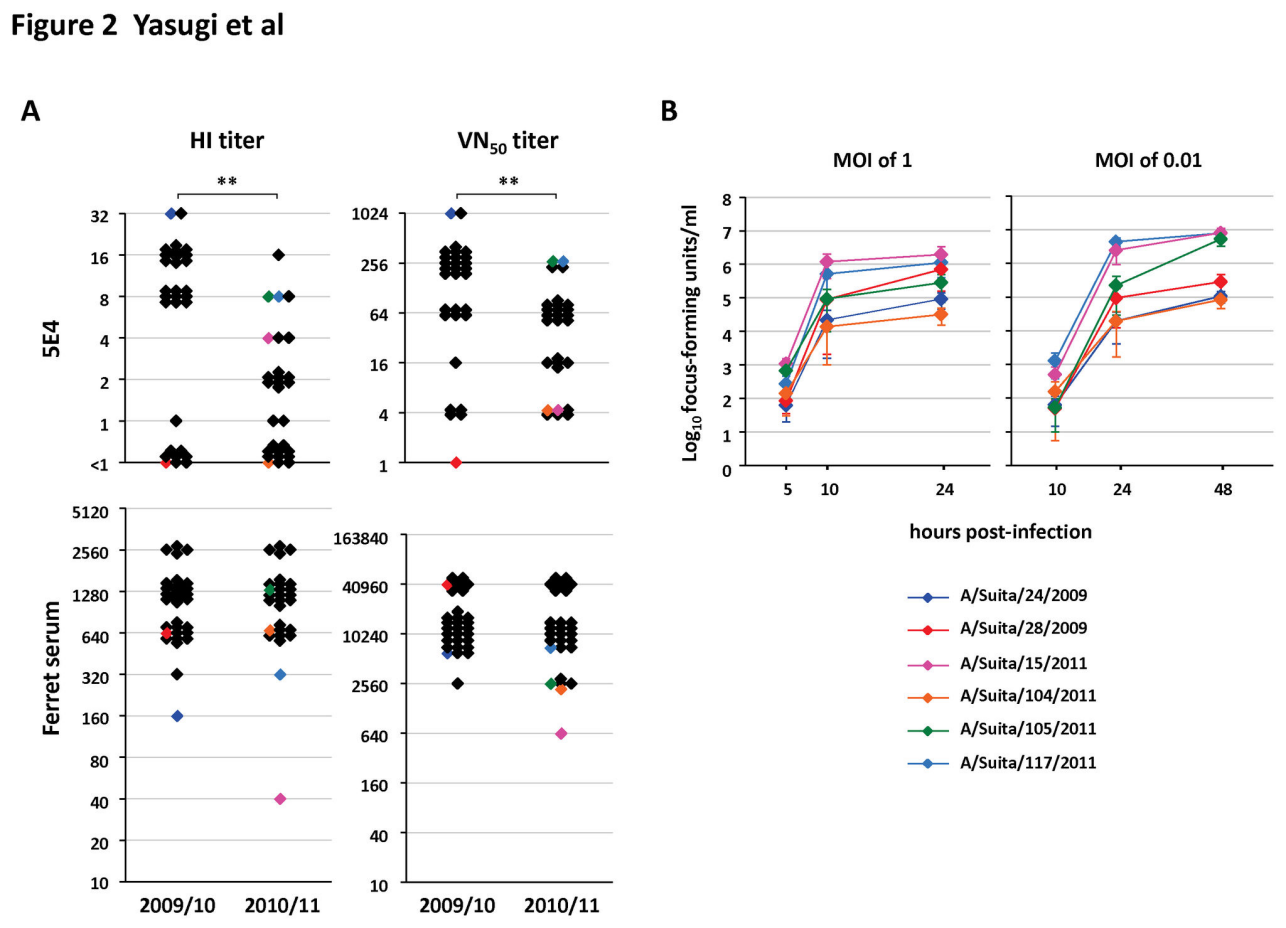
The reactivity of viral isolates in Japan to HuMAb 5E4 and anti-A(H1N1)pdm09 ferret serum. (A) Thirty and twenty-eight viral isolates obtained in the 2009/10 and 2010/11 winter seasons, respectively, in Osaka, Japan were titrated for HI and VN_50_ activities using 5E4 and ferret serum. Colored dots correspond to the individual viral isolates for which kinetic data are shown in Figure 2B. The y-axis shows the reciprocal antibody dilutions. Asterisks denote *P*<0.01. (B) Viral replication kinetics were examined in six randomly selected viral isolates. MDCK cells were infected with the isolates at a multiplicity of infection (MOI) of 1 (left panel) or 0.01 (right panel). At 5, 10, 24 and 48 hours post-infection, the supernatant was titrated by focus-forming assay.

The emergence of possible antigenic variants of Sb during the period between 2009 and 2011 was investigated by testing for loss of reactivity with 5E4 in HI and VN tests and comparing this with the reactivity with ferret anti-A(H1N1)pdm09 serum (Figures 2A and S2, and Tables S1 and S2). The assays were performed against the 30 and 28 isolates of A(H1N1)pdm09 that were obtained from patients’ nasal swabs in December 2009 (2009/10 season) and January 2011 (2010/11 season), respectively, in Osaka, Japan. In the 2009/10 season, 5E4 had bimodal peaks in HI titers, with one group showing less than 1 and the other showing 8 to 32. The reactivity decreased significantly in the 2010/11 season and most of the isolates showed quite low HI titers; less than 2. Meanwhile, the ferret serum showed 160 to 2,560 HI titers and mean values were distributed between 640 and 1,280 in both periods. In the VN_50_ assay, 5E4 showed peak VN_50_ titers of 256 in the 2009/10 season. The reactivity of 5E4 decreased significantly in the 2010/11 season and the peak VN_50_ titers at this time were 64. By contrast, ferret serum showed similar titers in 2,560 to 40,960 in both periods. These results indicated that the antigenic variants in Sb increased significantly in Japan during 2009 and 2011. Six isolates, A/Suita/24/2009, A/Suita/28/2009, A/Suita/15/2011, A/Suita/104/2011, A/Suita/105/2011 and A/Suita/117/2011, which showed various reactivities against 5E4 and ferret serum, were randomly extracted (as indicated by colored dots in Figure 2A) and viral replication kinetics were examined. MDCK cells were infected with each of these viruses at a multiplicity of infection (MOI) of 1 or 0.01. At 5, 10, 24, and 48 hours post-infection, the supernatants were collected for infectivity titration of progeny virus. As shown in Figure 2B, these six viral isolates replicated with various kinetics and did not show a correlation between reactivity against antibodies and viral growth. These results suggest that strains that have low reactivity against 5E4 could have a replication advantage under the conditions of host 5E4-like immune pressure.

### Substitution rate of H1N1pdm isolated in Japan

**Figure 3:**
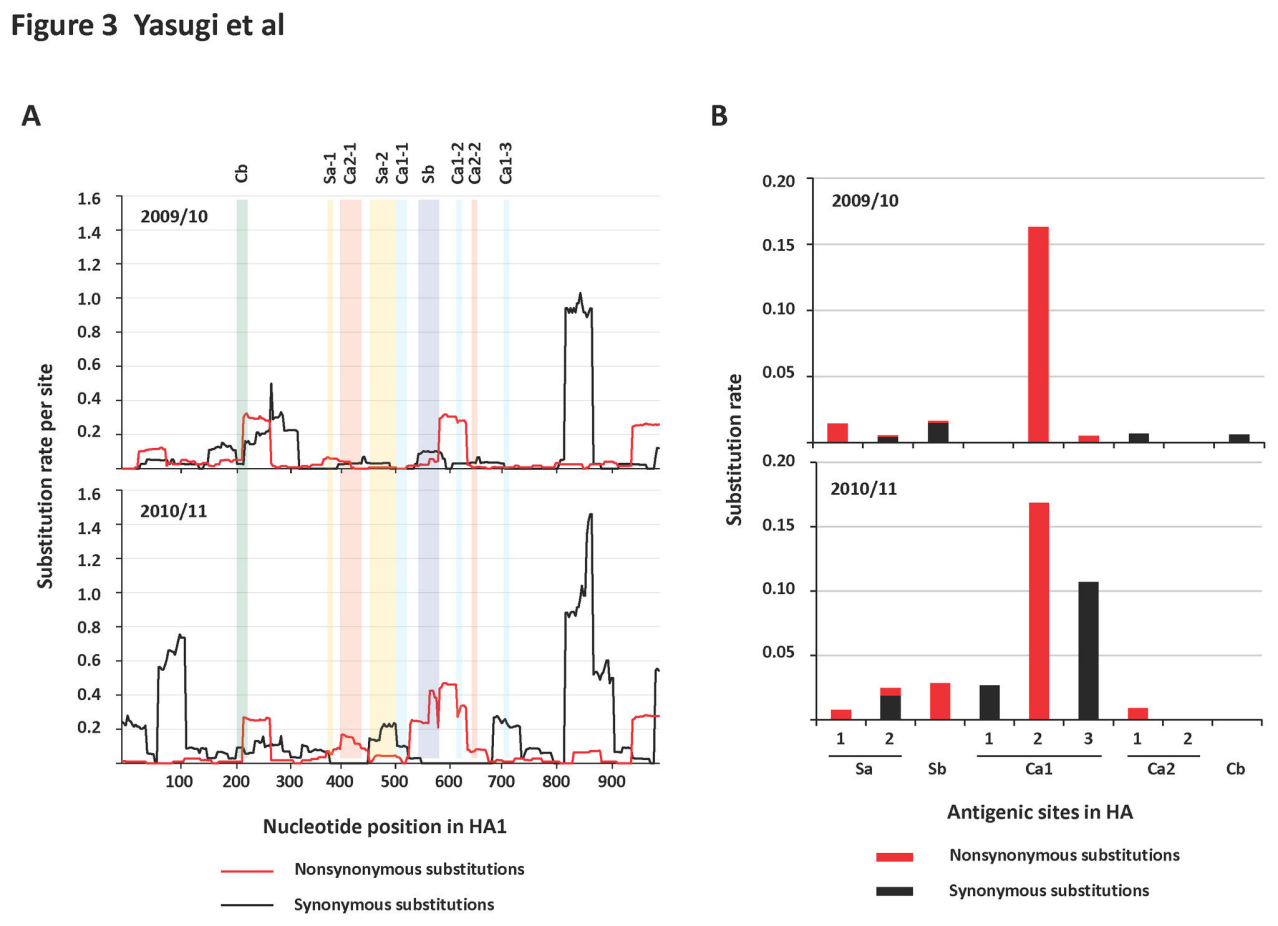
The rates of nonsynonymous and synonymous substitutions of the coding region of HA1 derived from viral isolates during 2009 to 2011 in Japan. (A) The HA1 gene in the 58 viral strains for which data are shown in Figure 2A was direct-sequenced and aligned with HA1 in A/California/7/2009 as a control. The rates of nonsynonymous and synonymous substitutions of the coding region of the HA1 gene were calculated for each 51 bp window (sliding in 3 bp increments) and are indicated by red and black colors, respectively. Colored bars mark the antigenic sites, as follows: Sa (Sa-1 and -2) in yellow, Sb in blue, Ca1 (Ca1-1, -2 and -3) in cyan, Ca2 (Ca2-1 and -2) in orange and Cb in green. (B) The nonsynonymous (red) and synonymous (black) substitution rates within nine regions of five antigenic sites were also calculated.

Next, the genetics of the emerging HA variants in A(H1N1)pdm09 in Osaka, Japan was investigated. All of the viral isolates used in Figure 2A were subjected to RNA extraction, one-step RT-PCR, and direct sequencing of the HA gene. To estimate the frequency of genetic mutations, the rates of synonymous (*d*_S_) and nonsynonymous (*d*_N_) substitutions were calculated per site in the HA1 gene against the control vaccine strain, A/California/7/2009, by the Miyata-Yasunaga method [31] using Sqdif Plot (Figure 3A). The substitution rates showed *d*_N_>*d*_S_ within nucleotide positions 210 to 260, 580 to 630, and 920 to 980 in both the 2009/10 and 2010/11 seasons, suggesting that these substitutions are caused by positive selection in 2009. By contrast, positions 380 to 450, 520 to 580, and 630 to 680 showed *d*_N_>*d*_S_ only in the 2010/11 season, indicating that positive selection occurred in these positions after the 2009/10 season. We focused the substitution rates at five antigenic sites (Figure 3B). Sa and Ca1 showed *d*_N_>*d*_S_ in the 2009/10 season, suggesting that these antigenic sites underwent positive selection when compared to A/California/7/2009, one of the earliest viruses isolated. Notably, Sb and Ca2 showed *d*_N_>*d*_S_ in the 2010/11 season, indicating that the mutations in Sb and Ca2 were caused by positive selection during the 2009/10 and 2010/11 seasons. Combined with the results shown in Figure 2, the data suggest that the antigenic variants of Sb emerged in Japan between 2009 and 2011 as a result of positive selection, including pressure exerted by the host immune response. 

### Diversity of amino acid sequences of the antigenic sites in the HA gene between April 2009 and January 2013 worldwide

**Figure 4:**
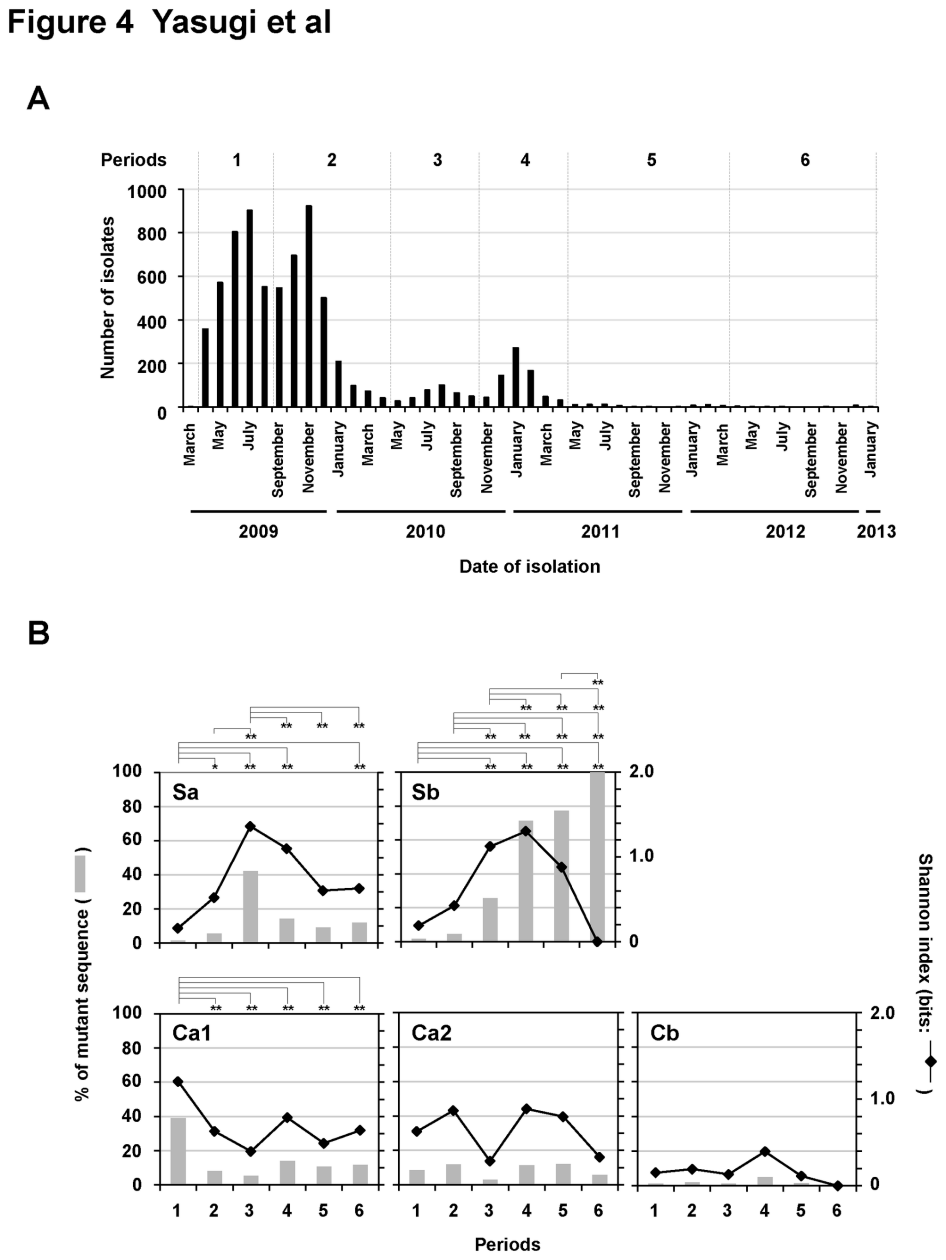
Genetic analysis of HA sequences of 7,415 A(H1N1)pdm09 isolates collected throughout the world, obtained from GenBank between April 2009 and January 2013. (A) The distribution of epidemics of A(H1N1)pdm09 globally. A total of 7,415 HA sequences of A(H1N1)pdm09 were aligned individually according to their date of isolation (between April 2009 and January 2013). They are divided into six periods: Periods 1 to 6. (B) The ratios of amino acid mutants (bar charts) and the Shannon indices (line charts) at each of the antigenic sites (Sa, Sb, Ca1, Ca2, and Cb) in the HA protein during Periods 1 to 6. The amino acid sequences in these antigenic sites were aligned and their mutation rates estimated (with respect to the dominant sequence in Period 1). *P<0.05 and **P<0.01.

To examine the genetic evolution of newly emerged A(H1N1)pdm09 in response to selective pressure, 7,415 HA genes in A(H1N1)pdm09 were extracted globally between April 2009 and January 2013 from the GenBank database. As shown in Figure 4A, there were four epidemic peaks for A(H1N1)pdm09: April to August 2009, September 2009 to April 2010, May to October 2010 and November 2010 to April 2011; these periods actually showed a good correlation with the rate of incidence of A(H1N1)pdm09 infection reported on FluNet (Global Circulation of Influenza Viruses; WHO) [43] and in the Infectious Agents Surveillance Report (National Institute of Infectious Diseases, Japan) [44]. These four peaks were designated Periods 1, 2, 3 and 4, respectively, as shown in Figure 4A. May 2011 to January 2013 was divided into two periods: Periods 5 and 6 (May 2011 to March 2012, and April 2012 to January 2013, respectively) (Figure 4A). The amino acid sequences of five antigenic sites, Ca1, Ca2, Cb, Sa and Sb, were individually aligned (Tables S3 to S7) and divided into two groups: the dominant sequence in Period 1 and mutant sequences for each antigenic site (Figure 4B). Genomic diversity was also calculated using the Shannon index (Figure 4B). The Shannon indices for Ca1, Ca2, Cb and Sa showed good correlation with the mutation rate. Mutations in Ca1 decreased significantly after Period 2, suggesting that the amino acid residues in Ca1 seem to become well-adapted for infection of humans. Ca2 and Cb showed quite low mutation rates throughout the experimental period. Both the mutation rate and diversity at the Sa epitope increased significantly after Period 2 and peaked in Period 3. Then the number of variants decreased and eventually the variants did not predominate. Notably, the number of mutants in Sb readily increased and exceeded that of the originally dominant sequence in Period 4. Ultimately, only variant sequences were detected in Period 6, although the number of isolates was low (Table S7). Diversity peaked in Period 4. Thus, globally, genetic variants against the antigenic site Sb have emerged significantly. 

## Discussion

In this study, characterization of viral isolates of A(H1N1)pdm09 using a neutralizing HuMAb, 5E4, revealed an unusually high rate of introduction of variants against the antigenic site Sb from 2009 to 2011 in Osaka, Japan. Also, 2010/11 isolates showed an increased rate of genetic mutation at Sb. In agreement with these findings, our global characterization of amino acid sequences at Sb derived from the GenBank database exhibited the same results. These results indicate that antigenic variants against Sb have emerged and have increased in prevalence. These findings may be of considerable utility for developing a future vaccine against A(H1N1)pdm09. 

Classical seasonal H1N1 has five antigenic sites in the globular head of the HA protein: Sa, Sb, Ca1, Ca2 and Cb. It has not been fully elucidated whether these antigenic sites are equally important for A(H1N1)pdm09. However, a similar antigenic structure to that of seasonal H1N1 has been predicted for A(H1N1)pdm09 by homology modeling [16]. Moreover, several MAbs have been established against the globular head, including Sa, Sb and Ca2 [17-19]. Thus, some of the classical antigenic sites in the HA molecule seem important for immunity against A(H1N1)pdm09. In this study, vaccination for A(H1N1)pdm09 elicited neutralizing antibodies in human, including 5E4 against Sb. Also, Sb exhibited a significant nonsynonymous substitution rate during 2009/10 and 2010/11 (Figure 3). Taken together, these data indicate that at least the antigenic site Sb has become one of the significant foci of mutation in A(H1N1)pdm09 due to selection pressure including host immune response. By contrast, it is unclear whether other antigenic sites are crucial for immunity against A(H1N1)pdm09 because we did not generate HuMAbs against the other antigenic sites. Further studies are needed to address the importance of other antigenic sites.

Genetically distinct variants containing several significant amino acid changes in both the HA and NA genes have emerged in 2010. Ding et al. reported that T206 and E/G225 had been genetically positively selected by April 2010. Notably, both amino acid residues are located in the highly variable epitope regions of HA1 [45]. First in Singapore, and subsequently in Australia and New Zealand, the genetic variants with dual HA (N128D and E377K) and NA (M15I and N189S) mutations predominated in H1N1pdm by June 2010, although at that point there were no major antigenic differences in any of the variant viruses [4]. In addition, a mutant with dual HA substitutions, at S188T and E377K, became the predominant strain in Hong Kong in 2010 [5]. The substitutions at T206, E/G225, D128, and T188 described above are located in the antigenic sites Ca1, Ca2, Sa, and Sb of HA, respectively. In this study, we analyzed genetic diversity on a global scale (Figure 4 and Tables S3 to S7) and characterized the samples from April 2009 to January 2013. Consistent with the reports described previously [4,5,45], T206 at Ca1 was dominant after Period 2, and the number of variants decreased significantly (Figure 4B and Table S3), suggesting that Ca1 underwent selection pressure during the early phase of the pandemic. In addition, the incidence of E/G225 at Ca2 and N128D at Sa increased during Periods 2 and 3, respectively; however, these mutations did not predominate, implying that they might have been associated with a disadvantage during A(H1N1)pdm09 replication. By contrast, the incidence of S188T at Sb increased markedly and completely replaced the original sequence during Period 6 (Table S7). It is difficult to determine whether the S188T substitution arose from antigenic selection or from adaptation using genetic analyses techniques alone. We also found that that antigenic variation at Sb was generated in Osaka, Japan, during 2009/10 and 2010/11 (Periods 2 and 4). Taken together, the results of the present study suggest that genetic variation in Sb may have been caused by antigenic selective pressure. 

We showed a high level of genetic variation in Ca2 and Sb in 2010/11 samples in Japan (Figure 3). The emergence of Sb antigenic variants was revealed using HuMAb 5E4. By contrast, it is not clear whether the emergence of the genetic variants in Ca2 was caused by selective pressure on Ca2 itself or by structural compensation for the mutations in Sb [46,47]. Four or more amino acid changes occurring in at least two of the five antigenic sites of HA are generally seen in significant antigenic variants [48,49]. Therefore, it is important to continue to survey the emergence of antigenic variants against A(H1N1)pdm09, including antigenic site Ca2.

In influenza surveillance, detailed antigenic characterization of isolates is usually performed with polyclonal ferret sera against influenza virus. However, while standard infected ferret sera react well, MAb reactivity with viral isolates can be heterogeneous [50]. In this study, reduced reactivity against HuMAb 5E4 was shown significantly in 2010/11 specimens in Japan even when infected ferret serum neutralized well against these viral strains. Thus, a longitudinal study is now required, using several HuMAbs and viral isolates, to gain a better understanding of viral antigenic evolution. This is indeed essential for the assessment of A(H1N1)pdm09 vaccination strategies.

## Supporting Information

Figure S1 **AC-ELISA using wild-type viruses (open bars) and escape mutants (solid bars) of A/Suita/1/2009 (left panel) and A/Osaka/63/2009 (right panel).** MuMAb C179 and HuMAb D23-1G7C2 were used as the coating and detecting antibodies, respectively. All data are represented as the means ± s.d. of three independent experiments.
(PDF)

Figure S2 **The distribution of HI and VN_50_ titers in 30 and 28 viral isolates obtained in 2009/10 and 2010/11, respectively, in Osaka, Japan using HuMAb 5E4 and ferret anti-H1N1pdm serum.** The X- and Y-axises show the reciprocal antibody dilutions. Colored dots indicate the individual viral isolates for which data are shown in Figure 2B.
(PDF)

Table S1 **VN_50_ and HI titers by HuMAb 5E4 and ferret serum against viral isolates in 2009/2010.**
(PDF)

Table S2 **VN_50_ and HI titers by HuMAb 5E4 and ferret serum against viral isolates in 2010/2011.**
(PDF)

Table S3 **The diversity of the amino acid residues in the antigenic site Ca1 in Periods 1 to 6.**
(PDF)

Table S4 **The diversity of the amino acid residues in the antigenic site Ca2 in Periods 1 to 6.**
(PDF)

Table S5 **The diversity of the amino acid residues in the antigenic site Cb in Periods 1 to 6.**
(PDF)

Table S6 **The diversity of the amino acid residues in the antigenic site Sa in Periods 1 to 6.**
(PDF)

Table S7 **The diversity of the amino acid residues in the antigenic site Sb in Periods 1 to 6.**
(PDF)
