# Supplementary figures and images for: Emerging Antigenic Variants at the Antigenic Site Sb in Pandemic A(H1N1)2009 Influenza Virus in Japan Detected by a Human Monoclonal Antibody
Source: PLoS One. 2013 Oct 16;8(10):e77892. doi: 10.1371/journal.pone.0077892 (PMC3797713; doi:10.1371/journal.pone.0077892)

Figure S1 Yasugi et al

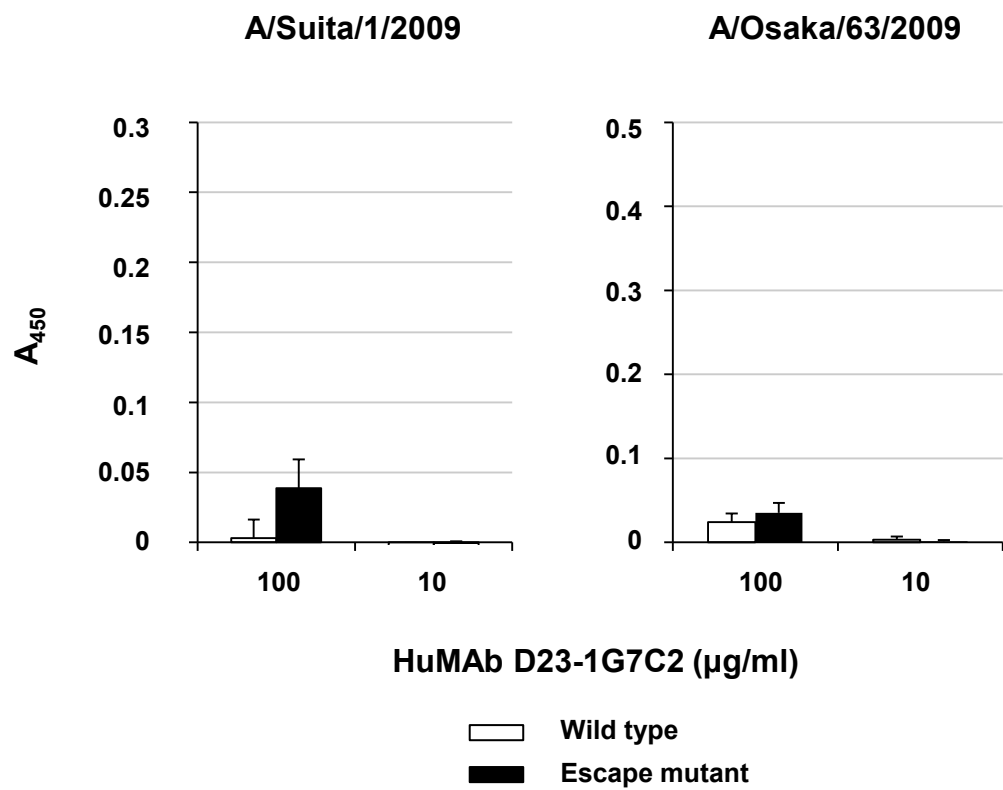

Supplement: Figure S1 — AC-ELISA using wild-type viruses (open bars) and escape mutants (solid bars) of A/Suita/1/2009 (left panel) and A/Osaka/63/2009 (right panel). MuMAb C179 and HuMAb D23-1G7C2 were used as the coating and detecting antibodies, respectively. All data are represented as the means ± s.d. of three independent experiments. (PDF) [file pone.0077892.s001.pdf]

Figure S2 Yasugi et al

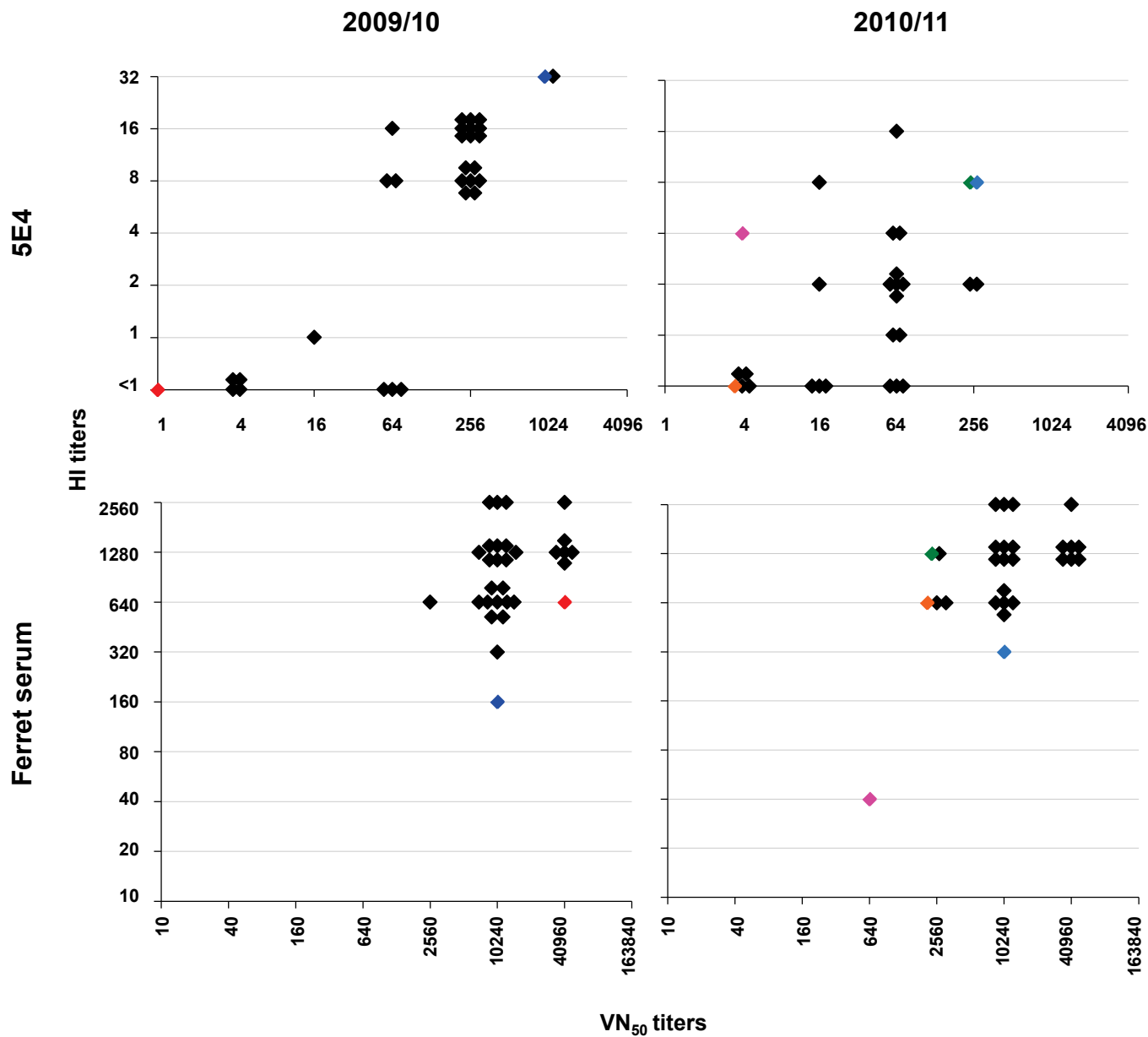

Supplement: Figure S2 — The distribution of HI and VN50 titers in 30 and 28 viral isolates obtained in 2009/10 and 2010/11, respectively, in Osaka, Japan using HuMAb 5E4 and ferret anti-H1N1pdm serum. The X- and Y-axises show the reciprocal antibody dilutions. Colored dots indicate the individual viral isolates for which data are shown in Figure 2B. (PDF) [file pone.0077892.s002.pdf]
