# Supplementary material for: Emerging Antigenic Variants at the Antigenic Site Sb in Pandemic A(H1N1)2009 Influenza Virus in Japan Detected by a Human Monoclonal Antibody
Source: PLoS One. 2013 Oct 16;8(10):e77892. doi: 10.1371/journal.pone.0077892 (PMC3797713; doi:10.1371/journal.pone.0077892)
Supplement: Table S1 — VN50 and HI titers by HuMAb 5E4 and ferret serum against viral isolates in 2009/2010. (PDF) [file pone.0077892.s003.pdf]

Supporting Tables

Table S1. VN_{50} and HI titers by HuMAb 5E4 and ferret serum against viral isolates in 2009/10.

2009/10	5E4		Ferret serum (×10)	
isolate	VN_{50}	HI	VN_{50}	HI
Suita1	64	16	1024	64
Suita3	256	16	1024	64
Suita4	64	8	1024	64
Suita5	256	16	1024	128
Suita6	1024	32	4096	128
Suita7	64	<1	1024	64
Suita8	256	8	1024	64
Suita9	256	16	4096	256
Suita10	4	<1	1024	128
Suita11	64	<1	1024	64
Suita12	4	<1	1024	128
Suita13	256	8	1024	64
Suita14	256	8	4096	128
Suita15	256	16	4096	128
Suita16	64	8	1024	256
Suita20	256	8	1024	64
Suita21	256	16	1024	256
Suita23	256	16	4096	128
Suita24	1024	32	1024	16
Suita25	256	16	1024	256
Suita26	256	8	1024	128
Suita27	256	16	4096	128
Suita28	1	<1	4096	64
Suita29	256	8	1024	128
Suita30	4	<1	1024	128
Suita31	4	<1	256	64
Suita32	16	1	1024	32
Suita33	256	8	1024	64
Suita34	256	16	1024	128
Suita35	64	<1	1024	128
