# Supplementary material for: Emerging Antigenic Variants at the Antigenic Site Sb in Pandemic A(H1N1)2009 Influenza Virus in Japan Detected by a Human Monoclonal Antibody
Source: PLoS One. 2013 Oct 16;8(10):e77892. doi: 10.1371/journal.pone.0077892 (PMC3797713; doi:10.1371/journal.pone.0077892)
Supplement: Table S2 — VN50 and HI titers by HuMAb 5E4 and ferret serum against viral isolates in 2010/2011. (PDF) [file pone.0077892.s004.pdf]

Table S2. VN_{50} and HI titers by HuMAb 5E4 and ferret serum against viral isolates in 2010/11.

2010/11	5E4		Ferret serum (×10)	
isolate	VN_{50}	HI	VN_{50}	HI
Suita12	4	<1	4096	128
Suita15	4	4	64	4
Suita31	4	<1	1024	128
Suita46	16	8	1024	128
Suita83	64	<1	1024	64
Suita84	64	1	4096	128
Suita85	64	4	1024	128
Suita86	64	2	1024	64
Suita87	64	2	4096	256
Suita88	256	2	1024	64
Suita89	64	16	1024	64
Suita90	4	<1	256	128
Suita91	256	2	1024	128
Suita92	16	<1	256	64
Suita93	16	<1	1024	64
Suita94	64	4	4096	128
Suita95	64	<1	1024	256
Suita97	64	2	256	64
Suita99	64	1	4096	128
Suita100	16	2	1024	128
Suita101	64	2	1024	128
Suita103	64	<1	4096	128
Suita104	4	<1	256	64
Suita105	256	8	256	128
Suita106	64	2	4096	128
Suita107	16	<1	1024	256
Suita109	4	<1	1024	256
Suita117	256	8	1024	32
