# Supplementary material for: Emerging Antigenic Variants at the Antigenic Site Sb in Pandemic A(H1N1)2009 Influenza Virus in Japan Detected by a Human Monoclonal Antibody
Source: PLoS One. 2013 Oct 16;8(10):e77892. doi: 10.1371/journal.pone.0077892 (PMC3797713; doi:10.1371/journal.pone.0077892)
Supplement: Table S3 — The diversity of the amino acid residues in the antigenic site Ca1 in Periods 1 to 6. (PDF) [file pone.0077892.s005.pdf]

Table S3. The diversity of the amino acid residues in the antigenic site Ca1 in Periods 1 to 6.

											Period					
											#1	#2	#3	#4	#5	#6
I	N	D	K	G	T	S	R	E	P	G	1940	2834	340	606	59	15
-	-	-	-	-	S	-	-	-	-	-	1147	142	2	4		
-	-	-	-	-	-	-	K	-	-	-	39	21	12	76	7	
-	-	-	-	-	S	-	K	-	-	-	27	8				
-	-	N	-	-	-	-	-	-	-	-	4	5				
V	-	-	-	-	-	-	-	-	-	-	3	3				
-	-	-	-	-	X	-	-	-	-	-	3	3				
-	-	-	-	-	-	-	S	-	-	-	2					
-	-	-	-	-	-	-	-	G	-	-	2	6				
-	-	-	-	E	-	-	-	-	-	-	2	10				
-	-	-	-	R	-	-	K	-	-	-	2					
-	-	-	-	-	-	-	-	-	S	-	2	1				
-	X	X	-	-	S	-	-	-	-	-	1					
-	-	-	R	-	S	-	-	-	-	-	1					
V	-	-	-	-	S	-	-	-	-	-	1					
-	-	-	-	-	-	-	-	K	-	-	1	15	1	5		
-	-	-	-	R	S	-	-	-	-	-	1	1				
-	-	-	-	-	-	-	-	-	-	R	1					1
-	-	-	-	-	A	-	-	-	-	-	1	1				
-	-	-	-	-	S	-	-	K	-	-	1					
-	-	-	-	R	-	-	-	-	-	-	1		2	3		1
-	-	-	-	-	-	-	K	K	-	-	1			2		
-	-	-	-	-	X	X	X	-	-	-	1	1				
-	-	-	-	-	-	-	-	A	-	-	1			2		
-	-	-	-	-	-	-	-	D	-	-		7				
-	-	R	-	-	-	-	-	-	-	-		4				
-	-	-	R	-	-	-	-	-	-	-		4				
-	-	-	-	-	-	-	G	-	-	-		3				
T	-	-	-	-	-	-	-	-	-	-		2				
-	-	-	-	X	-	-	-	-	-	-		2				
-	-	-	-	-	-	-	-	V	-	-		2				
-	H	-	-	-	-	-	-	-	L	-		1				
L	-	-	-	-	-	-	-	-	-	-		1				
-	-	-	-	-	L	-	-	-	-	-		1				
-	-	-	-	-	-	-	T	-	-	-		1				
-	-	-	T	E	-	-	K	-	-	-		1				
-	-	-	-	E	S	-	-	-	-	-		1				
-	-	-	-	-	-	-	-	Q	-	-		1				
F	-	-	-	-	-	-	-	-	-	-		1				
-	-	-	-	-	-	-	X	-	-	-		1	1			
-	-	-	E	-	-	-	-	-	-	-			1			
-	-	-	-	-	-	T	-	-	-	-				1		
-	L	-	-	-	-	-	-	-	-	-				1		
-	V	-	-	-	-	-	-	-	-	-				1		
M	-	-	-	-	-	-	-	-	-	-				1		
S	-	-	-	-	-	-	-	-	-	-				1		
-	-	-	-	-	-	-	E	-	-	-				1		
total											3185	3084	359	704	66	17
