# Supplementary material for: Emerging Antigenic Variants at the Antigenic Site Sb in Pandemic A(H1N1)2009 Influenza Virus in Japan Detected by a Human Monoclonal Antibody
Source: PLoS One. 2013 Oct 16;8(10):e77892. doi: 10.1371/journal.pone.0077892 (PMC3797713; doi:10.1371/journal.pone.0077892)
Supplement: Table S4 — The diversity of the amino acid residues in the antigenic site Ca2 in Periods 1 to 6. (PDF) [file pone.0077892.s006.pdf]

Table S4. The diversity of the amino acid residues in the antigenic site Ca2 in Periods 1 to 6.

									Period					
									#1	#2	#3	#4	#5	#6
C	P	H	A	G	A	K	R	D	2914	2714	348	624	58	16
-	-	-	-	-	-	-	-	E	160	194				
-	-	-	-	-	-	-	-	G	26	59	3	13	1	1
-	S	-	-	-	-	-	-	-	23	3			1	
-	-	-	-	-	-	-	-	X	17	18		6		
-	L	-	-	-	-	-	-	-	13	8				
-	-	-	-	-	-	-	-	N	7	15		12		
-	-	-	-	-	-	-	X	-	3					
-	-	-	-	-	V	-	-	-	2	1		1		
-	S	-	-	-	-	-	-	E	2	8				
-	-	-	-	-	-	-	-	B	2	1		3		
-	-	-	D	-	-	-	-	-	2	12	3			
-	-	Y	-	-	-	-	-	-	1	5				
-	-	-	X	X	-	-	-	-	1					
-	-	-	-	E	-	-	-	G	1					
X	-	-	-	-	-	-	X	X	1					
-	-	Q	-	-	-	-	-	-	1	1		21	3	
-	-	-	-	-	-	-	X	X	1					
-	-	-	-	-	T	-	-	-	1	5				
-	-	-	-	E	-	-	-	-	1	4		1		
-	-	-	-	-	-	-	G	-	1	1				
-	-	L	-	-	-	-	-	G	1					
-	-	R	-	-	-	-	-	-	1	1			3	
-	-	-	-	-	-	R	-	-	1	8	1			
X	X	-	-	-	-	-	X	X	1					
-	-	N	-	-	-	-	-	-	1	3		1		
-	-	-	-	-	E	-	-	-		2	1	8		
-	-	-	T	-	-	-	-	-		2	1	2		
-	-	-	S	-	-	-	-	-		2				
-	-	-	V	-	-	-	-	G		1		1		
-	-	-	-	-	-	-	-	A		1		1		
-	-	-	-	-	-	-	M	-		1				
-	S	-	-	-	-	-	-	G		1				
-	T	-	-	-	-	-	-	-		3				
-	-	-	-	-	-	-	K	E		1				
-	-	-	-	-	-	-	Q	-						
-	-	-	V	-	-	-	-	-				5		
-	-	-	-	-	T	-	-	N						
-	-	-	-	-	-	-	K	X		2				
-	-	-	-	-	S	-	-	-		2		1		
-	X	-	-	-	-	-	X	-		1				
-	-	-	-	A	-	-	-	-		1				
-	-	-	D	-	-	-	-	G		1	1			
-	X	-	-	-	-	-	-	-		1				
-	-	-	-	-	T	-	-	E		1				
-	-	-	-	-	V	-	K	-			1			
-	-	-	V	-	-	-	-	X				2		
-	-	-	-	-	-	-	K	-				1		
-	-	-	T	-	-	-	-	G				1		
-	H	-	-	-	-	-	-	-					1	
total									3185	3084	359	704	66	17
