# Supplementary material for: Emerging Antigenic Variants at the Antigenic Site Sb in Pandemic A(H1N1)2009 Influenza Virus in Japan Detected by a Human Monoclonal Antibody
Source: PLoS One. 2013 Oct 16;8(10):e77892. doi: 10.1371/journal.pone.0077892 (PMC3797713; doi:10.1371/journal.pone.0077892)
Supplement: Table S5 — The diversity of the amino acid residues in the antigenic site Cb in Periods 1 to 6. (PDF) [file pone.0077892.s007.pdf]

Table S5. The diversity of the amino acid residues in the antigenic site Cb in Periods 1 to 6.

							Period					
							#1	#2	#3	#4	#5	#6
	L	S	T	A	S	S	3142	3028	354	69	65	17
	-	-	-	T	-	-	12	5		1		
	F	-	-	S	-	-	6	16				
	-	-	-	-	N	-	4	5	2	14		
	-	-	-	-	I	-	3	4				
	-	-	-	V	-	-	3					
	-	Y	-	-	-	-	2					
	-	-	K	-	-	-	2	2				
	-	-	X	-	-	-	1					
	-	P	-	-	-	-	1					
	-	-	S	-	-	-	1					
	I	-	-	-	-	-	1	2				
	-	-	-	-	G	-	1	2		1		
	-	-	-	E	-	-	1	1				
	-	-	-	-	R	-	1	5		1		
	-	F	-	-	-	-	1	1		1		
	-	-	-	-	-	L	1			1		
	X	-	-	-	-	-	1					
	-	-	-	X	-	-	1					
	-	-	I	-	-	-		5	1			
	-	-	-	S	-	-		4		1		
	F	-	-	T	-	-		1				
	-	T	-	-	-	-		1			1	
	-	-	-	-	X	X		1				
	-	-	X	X	X	X		1				
	F	-	-	-	-	-			1	13		
	-	A	-	-	-	-			1	1		
	-	-	-	-	-	P				1		
total							3185	3084	359	704	66	17
