# Supplementary material for: Emerging Antigenic Variants at the Antigenic Site Sb in Pandemic A(H1N1)2009 Influenza Virus in Japan Detected by a Human Monoclonal Antibody
Source: PLoS One. 2013 Oct 16;8(10):e77892. doi: 10.1371/journal.pone.0077892 (PMC3797713; doi:10.1371/journal.pone.0077892)
Supplement: Table S6 — The diversity of the amino acid residues in the antigenic site Sa in Periods 1 to 6. (PDF) [file pone.0077892.s008.pdf]

Table S6. The diversity of the amino acid residues in the antigenic site Sa in Periods 1 to 6.

															Period					
															#1	#2	#3	#4	#5	#6
P	N	K	K	G	N	S	Y	P	K	L	S	K	S	Y	3139	2915	208	604	60	15
-	-	-	-	E	-	-	-	-	-	-	-	-	-	-	6	19	1	2		
-	-	-	-	-	-	-	-	-	-	F	-	-	-	-	5					
-	-	-	-	-	-	-	-	-	-	-	N	-	-	-	5	32	2	20	1	
-	-	-	-	-	-	-	-	-	-	-	-	E	-	-	3	2				1
-	-	-	-	X	-	-	-	-	-	-	-	-	-	-	3					
-	-	-	-	-	X	-	-	-	-	-	-	-	-	-	2	1				
-	-	-	-	-	B	-	-	-	-	-	-	-	-	-	2					
-	-	-	-	-	-	-	-	-	-	-	I	-	-	-	2			1	2	
-	-	-	E	-	-	-	-	-	-	-	-	-	-	-	1	1	1	3		
-	-	-	Q	-	-	-	-	-	-	-	-	-	-	-	1	1				
-	-	-	-	-	S	-	-	-	-	-	-	-	-	-	1	2		1		
-	-	-	-	-	-	-	-	-	-	-	-	X	-	-	1	1				
-	-	-	-	-	K	-	-	-	-	-	-	-	-	-	1	7				
-	-	-	-	-	D	-	-	-	-	-	-	-	-	-	1	2		3		
X	-	-	-	-	-	-	-	-	-	-	-	-	-	-	1	1				
-	-	-	-	-	-	-	-	-	-	I	-	-	-	-	1	3		2		
S	-	-	-	-	-	-	-	-	-	-	-	-	-	-	1					
-	I	-	-	-	-	P	-	-	-	-	T	-	-	-	1					
-	-	-	-	-	-	-	-	-	E	-	-	-	-	-	1					
-	-	E	-	-	-	-	-	-	-	-	-	-	-	-	1			1		
-	-	-	-	-	T	-	-	-	-	-	-	-	-	-	1			1		
-	-	-	-	-	-	-	-	-	-	-	R	-	-	-	1	7				
-	-	-	T	-	-	-	-	-	-	-	-	-	-	-	1			2		
-	-	-	-	-	-	-	-	-	-	-	-	-	F	-	1	1		1		
-	-	-	-	-	-	-	-	-	M	-	-	-	-	-	1	2				
-	-	-	X	-	-	-	-	-	-	-	-	-	-	-	1			2		
-	D	-	-	-	-	-	-	-	-	-	-	-	-	-		40	132	26		
-	-	-	-	-	-	-	-	-	-	-	-	N	-	-		6	3	2		
-	-	-	-	-	-	-	-	-	R	-	-	-	-	-		4	1			1
-	-	-	-	-	-	-	-	-	-	I	-	N	-	-		3				
-	-	-	-	X	-	-	-	-	-	-	N	-	-	-		3				
-	-	-	-	-	-	L	-	-	-	-	-	-	-	-		3				
-	-	-	-	-	-	T	-	-	-	-	-	-	-	-		3				
-	-	-	-	R	-	-	-	-	-	-	-	-	-	-		2				
-	-	-	-	-	-	-	-	S	-	-	-	-	-	-		2				
-	-	-	-	-	-	-	-	-	T	-	-	-	-	-		2				
-	-	-	-	-	-	-	-	-	-	-	-	-	Y	-		2				
-	-	-	-	-	-	-	F	S	-	-	-	-	T	-		1				
-	-	-	-	R	-	-	-	-	-	-	-	Q	-	-		1				
X	X	-	-	-	-	-	-	-	-	-	-	-	-	-		1				
-	S	-	-	-	-	-	-	-	-	-	-	-	-	-		1		3		
X	X	-	-	-	-	-	-	-	-	-	N	-	-	-		1				
-	X	-	-	X	X	-	-	-	-	-	N	-	-	-		1				
-	-	-	-	X	X	-	-	-	-	-	-	-	-	-		1				
-	-	-	-	-	-	-	-	-	-	-	-	Q	-	-		1	1	1		
-	-	-	-	-	-	-	-	-	-	V	-	-	-	-		1				
-	-	-	-	-	-	-	-	-	-	-	-	I	-	-		1		2		
-	I	-	-	-	-	-	-	-	-	-	-	-	-	-		1				

	-	H	-	-	-	-	-	-	-	-	-	-	-	-	-		1				
	-	-	-	-	-	-	-	-	-	-	-	-	R	-	-		1		1	1	
	-	D	-	-	-	K	-	-	-	-	-	-	-	-	-		1	1			
	-	-	-	-	-	-	-	-	-	-	-	-	-	P	-		1				
	-	-	-	-	-	-	-	-	-	-	-	-	T	-	-		1	6	15	2	
	-	-	-	-	-	-	-	-	-	-	-	-	-	-	V		1		2		
	-	D	-	-	-	-	-	-	S	-	-	-	-	-	-			2			
	S	D	-	-	-	-	-	-	-	-	-	-	-	-	-			1			
	-	-	-	-	-	-	-	-	-	-	-	N	T	-	-				3		
	-	-	N	-	-	-	-	-	-	-	-	-	-	-	-				1		
	-	-	-	-	-	-	-	-	-	-	-	-	S	-	-				1		
	-	-	-	T	-	-	-	-	S	-	-	-	-	-	-				1		
	-	-	-	-	-	-	-	-	-	R	-	-	T	-	-				1		
	-	-	-	N	-	-	-	-	-	-	-	N	T	-	-				1		
	-	-	-	-	-	-	-	-	-	-	-	N	I	-	-				1		
total																3185	3084	359	704	66	17
