# Supplementary material for: Emerging Antigenic Variants at the Antigenic Site Sb in Pandemic A(H1N1)2009 Influenza Virus in Japan Detected by a Human Monoclonal Antibody
Source: PLoS One. 2013 Oct 16;8(10):e77892. doi: 10.1371/journal.pone.0077892 (PMC3797713; doi:10.1371/journal.pone.0077892)
Supplement: Table S7 — The diversity of the amino acid residues in the antigenic site Sb in Periods 1 to 6. (PDF) [file pone.0077892.s009.pdf]

Table S7. The diversity of the amino acid residues in the antigenic site Sb in Periods 1 to 6.

													Period					
													#1	#2	#3	#4	#5	#6
	T	S	A	D	Q	Q	S	L	Y	Q	N	A	3131	2942	267	202	15	
	-	N	-	-	-	-	-	-	-	-	-	-	10	13		1		
	-	-	-	-	-	-	-	I	-	-	-	-	6	11		4		
	-	-	-	-	-	-	N	-	-	-	-	-	6	4	3			
	-	-	-	-	-	-	-	J	-	-	-	-	4					
	-	I	-	-	-	-	-	-	-	-	-	-	4	6		9		
	X	-	-	-	-	-	-	-	-	-	-	-	3					
	-	-	-	-	-	-	-	-	-	-	T	-	3					
	-	-	-	-	-	-	-	-	-	-	-	V	2	7				
	-	-	T	-	-	-	-	-	-	-	-	-	2	60	2	4		
	-	-	-	-	-	-	R	-	-	-	-	-	2		1	2		
	-	-	-	-	-	-	G	-	-	-	-	-	2	5	1		1	
	-	-	-	X	-	-	-	-	-	-	-	-	2					
	-	-	-	-	R	-	-	-	-	-	-	-	1	1				
	-	R	-	-	L	-	-	I	N	-	-	-	1					
	-	-	-	-	-	-	X	-	-	-	-	-	1	1				
	A	-	-	-	-	-	-	-	-	-	-	-	1	2				
	-	T	-	-	-	-	-	-	-	-	-	-	1	1	77	463	50	17
	-	-	-	G	-	-	-	-	-	-	-	-	1	4	1	1		
	-	X	-	-	-	-	-	-	-	-	-	-	1					
	P	M	-	-	-	-	-	-	-	K	-	-	1					
	-	-	V	-	-	-	-	-	-	-	-	-		5				
	-	-	-	-	-	-	-	-	-	-	S	-		3				
	-	-	X	-	-	-	-	-	-	-	-	-		3				
	-	-	-	-	-	-	-	-	-	-	-	T		3				
	-	-	-	A	-	-	-	-	-	-	-	-		1				
	-	-	-	-	X	X	X	X	X	X	X	X		1				
	X	-	-	-	-	-	-	-	X	-	X	-		1				
	-	-	-	-	X	X	-	-	-	-	-	-		1				
	-	X	X	X	X	X	X	X	X	X	X	-		1				
	-	-	-	-	-	-	-	F	-	-	-	-		1				
	N	-	-	-	-	-	-	-	-	-	-	-		1		1		
	S	-	-	-	-	-	-	-	-	-	-	-		1				
	I	-	-	-	-	-	-	-	-	-	-	-		1				
	-	-	-	-	-	-	-	-	-	-	D	-		1				
	-	-	-	-	-	-	-	-	-	-	-	E		1				
	-	-	S		-	-	-	-	-	-	-	-		1				
	-	-	D	G	-	-	-	-	-	-	-	-		1				
	-	-	-	-	-	-	-	-	-	-	-	T						
	-	T	T	-	-	-	-	-	-	-	T	-			2			
	-	T	-	-	-	-	-	-	-	-	S	-			1			
	-	T	-	-	-	-	I	-	-	-	-	-			1			
	-	T	-	-	-	-	-	I	-	-	-	-			1			
	-	-	T	-	-	-	-	-	-	-	-	E			1			
	-	T	-	-	-	-	N	-	-	-	-	-			1			
	-	S	T	-	-	-	-	-	-	-	-	-				12		
	-	-	-	-	-	-	I	-	-	-	-	-				3		
	-	S	-	-	-	-	-	-	-	-	S	-				1		
	-	-	G	-	-	-	-	-	-	-	-	-				1		
total													3185	3084	359	704	66	17
